# Development and Implementation of the World Health Organization Emergency Medical Teams: Minimum Technical Standards and Recommendations for Rehabilitation

**DOI:** 10.1371/currents.dis.76fd9ebfd8689469452cc8c0c0d7cdce

**Published:** 2018-07-09

**Authors:** Jody-Anne Mills, James Gosney, Fiona Stephenson, Peter Skelton, Ian Norton, Valerie Scherrer, Geraldine Jacquemin, Barbara Rau

**Affiliations:** Rehabilitation Team, Management of Noncommunicable Diseases, Disability, Violence and Injury Prevention, World Health Organization, Geneva, Switzerland; Eastern Virginia Medical School, Norfolk, United States of America; Service Manager, Livability Spinal Injury Centre, United Kingdom; Rehabilitation Project Manager, Handicap International United Kingdom, London; Emergency Operations and Partnerships, Emergency Operations, World Health Organization, Geneva, Switzerland; Independent rehabilitation and disability consultant, Belgium; Université de Montréal, Institut de Réadaptation Gingras-Lindsay de Montréal, Quebec, CanadaInstitut de Readaptation Gingras-Lindsay de Montreal; Technical Coordinator- Physiotherapy, International Committee of the Red Cross, Geneva, Switzerland

## Abstract

Emergency medical teams provide urgent medical and surgical care in emergencies characterized by a surge in trauma or disease. Rehabilitation has historically not been included in the acute phase of care, as teams have either not perceived it as their responsibility or have relied on external providers, including local services and international organizations, to provide services. Low- and middle-income countries, which often have limited rehabilitation capacity within their health system,  are particularly vulnerable to disaster and are usually  ill-equipped to address the increased burden of rehabilitation needs that arise. The resulting unmet needs for rehabilitation culminate in unnecessary complications for patients, delayed recovery, reduced functional outcomes, and often impede return to daily activities and life roles. Recognizing the systemic neglect of rehabilitation in global emergency medical response, the World Health Organization, in collaboration with key operational partners and experts, developed technical standards and recommendations for rehabilitation which are integrated into  the WHO verification  process for EMTs. This protocol report presents: 1) the rationale for the development of the standards and accompanying recommendations; 2) the methodology of the development process; 3) the minimum standards and other significant content included in the document; 4) challenges encountered during development and implementation; and 5) current and next steps to continue strengthening the inclusion of rehabilitation in emergency medical response.

## Introduction

The World Health Organization (WHO) Emergency Medical team (EMT) Initiative aims to professionalize and standardize emergency medical response through classification of EMTs into types 1, 2, and 3, and ‘specialized care teams’, based on service capacity (see Table 1). The classification is based on the publication, Classification and minimum technical standards for foreign medical teams[1] in sudden onset disaster 1, developed under the auspices of WHO in 2013. The minimum technical standards define the capacity of various team types against service capacity, length of stay, staff, response time, equipment, clinical service areas, and other factors. EMTs that wish to be included on WHO’s directory of Quality Assured Teams must be verified by WHO and peers against technical standards according to their type and adhere to guiding principles and core standards. Availability of classified, quality-assured teams enables countries to select international and national EMTs to respond who are best qualified to meet their emergency needs.

**Table 1: WHO classification of EMTs:** Table source: https://extranet.who.int/emt/sites/default/files/. Reprinted from (19) under a CC BY license, with permission from WHO Press, original copyright 2016. MINIMUM%20TECHNICAL%20STANDARDS.pdf

Type	Description	Capacity	Minimum length of stay
1 Mobile	Mobile outpatients teams: Teams to access the smallest communities in remote areas.	>50 outpatients a day	2 weeks
1 Fixed	Outpatient facilities with or without tented structure.	>100 outpatients a day	2 weeks
2	Inpatient facility with surgery.	>100 outpatients and 20 inpatients. 7 major and 15 minor operations a day	3 weeks
3	Referral level care, inpatient facilities, surgery and high dependency.	>100 outpatients and 40 inpatients, including 4-6 intensive care beds. 15 major and 30 minor operations a day.	4-6 weeks
Specialized care team	Teams that can join local facilities or EMTs to provide supplementary specialist care.	Variable	Variable

Rehabilitation, defined as “a set of interventions designed to optimize functioning and reduce disability…” 2 (p.35) constitutes an important aspect of care in the context of injury and trauma, and some communicable diseases. Rehabilitation interventions can target mobility, cognition, and self-care, among other domains of functioning to facilitate recovery and return to activity. Recognizing the systematic neglect of rehabilitation in emergency medical response and the impact of this on patient outcomes, the classification included rehabilitation as a standard for type 2 and 3 EMTs, stating, “Rehabilitation is one of the core functions of trauma care systems in regular healthcare and as such, FMTs [‘foreign medical teams’], should have specific plans for the provision of rehabilitation services to their patients post SOD [sudden-onset disaster]” 1 (p.78). Rehabilitation service requirements per team type were described generally but not specifically defined, however.

As the EMT initiative progressed towards the verification of teams in 2015, a need existed to further define the standards for rehabilitation, as well as those for ‘specialized care teams for rehabilitation’. It was also felt that EMTs would benefit additionally from recommendations on how to optimize patient outcomes through rehabilitation and effective systems of care; the recommendations thereby complement the standards in improving the quality of rehabilitative care provided. This protocol report presents the rationale for the development of the rehabilitation standards and the accompanying recommendations (hereafter referred to as ‘standards’), the methodology of the development process, and the standards themselves, as well as other significant content developed. Challenges encountered during development and implementation are discussed, as well as priority actions to further strengthen the inclusion of rehabilitation in emergency medical response.

[1] ‘Foreign medical teams’ are now termed ‘emergency medical teams’.

## Rationale

Several factors contribute to the historic neglect of rehabilitation by EMTs. Despite the benefits of early rehabilitation intervention being well documented, namely helping speed patient recovery and discharge, reducing complications and optimizing functional outcomes 2^, ^3^, ^4^, ^5^, ^6^, ^7^, ^8, rehabilitation is still at times perceived as a post-acute or long-term health strategy and a non-essential component of care. EMTs, long known for their role in delivering acute and life-saving interventions, have historically not prioritized the delivery of rehabilitation. Many have not recognized its necessity during the acute phase of care and consider it beyond their operational mandate. Many EMTs provided little or no rehabilitation to their patients following the earthquakes in Pakistan in 2005, Haiti in 2010, Philippines in 2013 and Nepal in 2015, for example. This situation, compounded by EMTs failing to effectively refer people to appropriate follow-up services post-discharge, and poor documentation and record keeping, resulted in considerable unmet needs for rehabilitation 9^, ^10^, ^11. The true impact of neglecting rehabilitation in emergency contexts is difficult to capture, due to the lack of standardized outcome measures to indicate changes in functioning, and partly because the full benefits of rehabilitation are realized longitudinally, beyond the departure of EMTs. Furthermore, in emergency contexts, in low- and middle-income countries in particular, rehabilitation research focused on functional outcomes is not prioritized, with resources concentrated on patient survival and care. Nevertheless, it can be safely assumed that the impact of failing to receive rehabilitation, particularly in relation to social and economic outcomes such as the ability to return to education or employment and improved independence, would be greatest amongst the economically disadvantaged- the same people likely to be most impacted by an emergency. Published disaster literature points to a growing awareness of the need for the integration of rehabilitation into the acute phase of response, and shows that patient outcomes have been compromised by the lack of early rehabilitation provision and follow-up 8^, ^10^, ^11^, ^12^, ^13^, ^14^, ^15^, ^16^, ^17.

## **Methodology**

The EMT minimum technical standards and recommendations for rehabilitation (hereon referred to as ‘the standards’) were informed by a systematic review of academic and grey literature, conducted in 2014. Embase and Pubmed databases were searched broadly with terms related to emergency medical teams and field hospitals, disability, impairment, policies, guidelines, and nongovernmental organizations. Additional references were obtained from citations in publications and relevant websites and were provided by members of the working group.

The working group was selected and convened by WHO and comprised of experts from key international organizations engaged in emergency response (see ‘Who’ in Table 2). Members were required to have operational experience in emergencies to ensure the standards reflected the austere environments in which care is delivered. Most rehabilitation experts who fit the criteria for inclusion were from Europe or North America. While limited geographical representation was not ideal, broad peer review from other regions served to mitigate limitations in perspective to some degree.

The working group convened at WHO headquarters in Geneva in June 2015 to develop an initial version of the standards document. Results of the evidence review, existing standards, and expert opinion of group members were considered. The alpha version was then disseminated for peer reviewer to global rehabilitation stakeholders, including international rehabilitation professional bodies, nongovernmental and international organizations and individual experts. Peer reviewer feedback was reviewed and compiled by the WHO Secretariat for consideration by the working group.

The working group re-convened in Geneva in August 2015 and integrated the peer review feedback into an updated version of the standards document, which was disseminated for a second round of peer review to stakeholders from the medical and surgical community, EMT coordinators, and humanitarian medical logisticians. Feedback was collated by the WHO Secretariat, and integrated into a pilot version of the standards document with input from the working group, which continued to liaise virtually.

The standards were piloted over several months during the verification of type 2 EMTs, resulting in improved clarification between ‘standards’ and ‘recommendations’, with several standards being changed to recommendations. The position of the standards as ‘minimum’ was also more strongly emphasised. With these changes incorporated, the standards document was endorsed by the EMT Strategic Advisory Group in 2016.

The finalized standards document was made publicly available[1] and the standards were integrated into the verification checklists for type 2 and 3 EMTs. Accessibility to the standards document was improved by its translation into Arabic, Chinese, French, German, Russian, and Spanish, and the creation of infographics [2]. Increasing awareness of the necessity of the standards occurred through the development of supporting communications materials including a brochure, poster [2] and video[3] that emphasizes the necessity of rehabilitation in emergencies. The standards were also presented at the 2016 Global EMT Meeting and other emergency and disaster forums.

**Table 2. Summary of the development and implementation of the EMT minimum technical standards and recommendations for rehabilitation**

**Figure:**
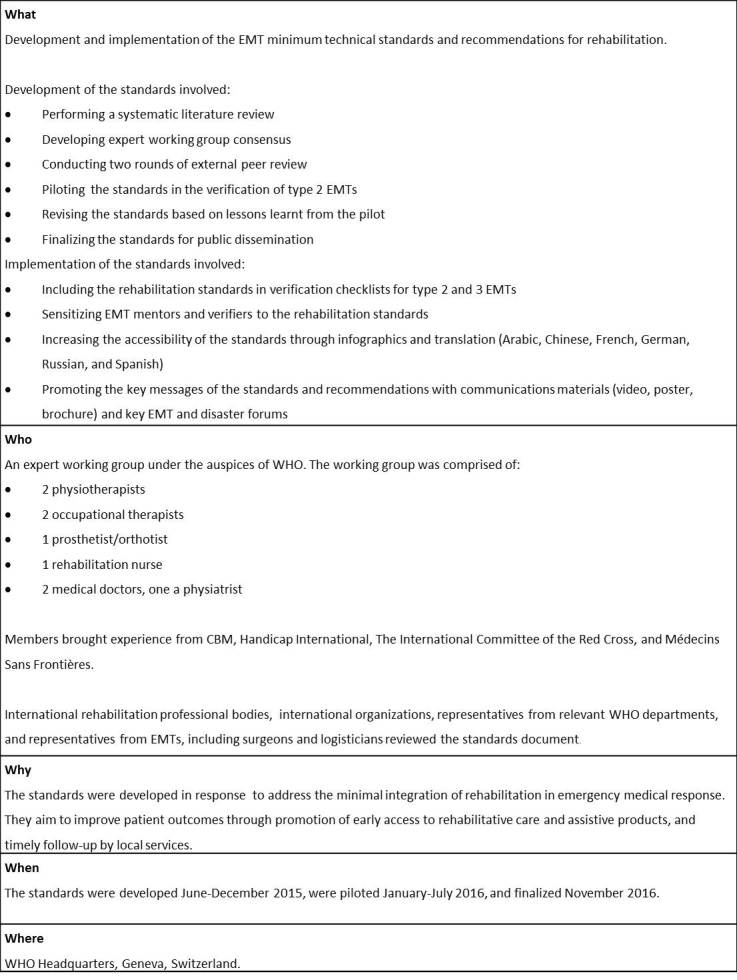

[1] https://extranet.who.int/emt/content/minimum-technical-standards-and-recommendations-rehabilitation

[2] These resources and others can be found at https://extranet.who.int/emt/resources

[3] Available on Youtube: https://www.youtube.com/watch?v=hW4tTr7buGw&t=107s

## Results

***The EMT minimum technical standards and recommendations for rehabilitation***

The standards document 18 contains technical standards and recommendations for EMTs, technical standards for ‘specialized care teams for rehabilitation’, considerations for rehabilitation in disease outbreaks, and additional content to support the delivery of rehabilitation in emergency response. Each of these components are detailed below.

The rehabilitation minimum technical standards for EMTs are based primarily on the context of sudden-onset disasters, including earthquakes, tsunamis or floods. They can be adapted for situations of conflict, which present a different pattern of injury and require longer length of stay for teams. The technical standards elaborate four seminal standards for type 2 and 3 EMTs:

1. at least one rehabilitation professional per 20 beds at the time of initial deployment, with further recruitment depending on case-load and local rehabilitation capacity;
2. allocation of a purpose-specific rehabilitation space of at least 12 m2 for all type 3 EMTs [1];
3. deployment of EMTs with at least the essential rehabilitation equipment and consumables according to type; and
4. if research is being undertaken during deployment, the EMT should maintain all professional, institutional and national ethical standards for research with human participants.

No minimum technical standards were developed for type 1 EMTs, who do not provide inpatient care, however the document does stipulate that they are required to have established referral mechanism for rehabilitation in place.

The recommendations are distinct from the technical standards in that they are not amenable to or required for demonstration for the purposes of verification, but are included to provide guidance on optimizing patient care. The recommendations relate to rehabilitation workforce, step-down facilities, rehabilitation equipment and consumables, field hospital accessibility, building rehabilitation capacity, information management and research in emergency response. The technical standards and recommendations are accompanied by a rationale for their inclusion, and details on how they can be demonstrated for verification, where appropriate.

‘Specialized care teams for rehabilitation’ are comprised of small groups of rehabilitation specialists that can be deployed to augment the rehabilitation capacity of an EMT or a local health facility. The standards require specialized care teams for rehabilitation to:

1. include at least three rehabilitation professionals of at least two disciplines, one of whom must be a physiotherapist;
2. ensure each member of the team has a least a bachelor degree or equivalent and at least three years of experience in trauma injury rehabilitation;
3. have the capacity to rapidly provide the listed essential equipment [2]; and
4. stay for the minimum length of stay of the EMT into which they embed, or for at least one month if they embed into a local health facility.

Drawing from lessons learnt in the West Africa Ebola outbreak 19, the document provides considerations for rehabilitation in outbreak response. These highlight the potential for those affected by a disease outbreak to have acute and long-term rehabilitation needs, such as for respiratory care or post-acute sequelae.

A proposed referral pathway, considerations for the management of patients with common traumatic injuries, a referral template to facilitate key information transfer, and an overview of rehabilitation input by EMT type and discharge considerations are also included in the document.

The document emphasises key messages for EMTs, notably that the early identification of rehabilitation needs and access to services improves outcomes, and close that coordination with local service providers facilitates a rehabilitative continuum of care that will support patients beyond the departure of EMTs. EMTs are also advised to not provide mobility devices, such as prostheses or wheelchairs, for long-term use, as these should ideally be provided locally to ensure access to ongoing maintenance and replacement when necessary.

***Implementation of the EMT minimum technical standards for rehabilitation in the verification of teams***

EMTs are verified by the WHO Secretariat and peers (members of other EMTs) using a checklist of standards for each team type. The technical standards for rehabilitation and guidance on how to demonstrate each standard are included on the checklist for teams to reference. The verification process includes a site visit when the EMT presents its full capability, including presentation of the essential rehabilitation equipment, designated rehabilitation space, and evidence of the inclusion of a qualified rehabilitation professional per 20 beds. Should a team have difficulty interpreting or meeting the standards, it can seek support from its designated EMT mentor[3], who in turn can seek technical advice from the WHO Secretariat. To date, WHO has verified four type 2 EMTs, and two type 1 EMTs, with many other teams currently undergoing the verification process. One specialized care team for rehabilitation has also been verified.

[1] Equipment in the essential lists include mobility devices, pressure relieving devices and various rehabilitation consumables. Refer to tables 4 and 7 in Emergency medical teams: Minimum technical standards and recommendations for rehabilitation for the complete lists: https://extranet.who.int/emt/sites/default/files/MINIMUM%20TECHNICAL%20STANDARDS.pdf.

[2] https://extranet.who.int/emt/page/understanding-emt-classification-process; see ' Step 3: Global Mentorship Program'

[3] https://extranet.who.int/emt/page/understanding-emt-classification-process; see ' Step 3: Global Mentorship Program'

## Discussion

The inclusion of standards for rehabilitation in the verification of EMTs is a significant step forward in terms of the quality and comprehensiveness of care EMTs deliver and represents increased emphasis on the longer-term functional outcomes of persons treated. The standards are particularly critical since countries vulnerable to disasters typically have limited institutional capacity to deliver quality rehabilitation. Several studies have indicated that only 26-55% of people in low- and middle-income countries receive the rehabilitation that they need 20. EMTs not providing rehabilitation or assuming local rehabilitation services will meet rehabilitation needs for their patients in an emergency , often characterized by degraded health services including rehabilitation, results in unmet rehabilitation needs. Benefits of collaborating with local services have generally not been fully realized in emergency response. Local rehabilitation providers understand the health and social systems and how to deliver context-appropriate interventions. They also ensure a rehabilitative continuum of care beyond the departure of EMTs.

Several challenging considerations were addressed by the working group in the development and implementation of the standards to ensure their value to emergency medical response and global relevance. First, the scientific evidence base for rehabilitation in the humanitarian context is lacking. Rehabilitation has historically not been prioritized in emergency medical response literature and, moreover, most accounts are sequestered within the grey literature as reports of operational rehabilitation service provider organizations. In the absence of strong evidence, development of the standards was based heavily on expert opinion. Working group membership represented different rehabilitation disciplines and types of international stakeholder organizations which yielded a range of relevant perspectives, broad awareness of current best practices, and significant experiential awareness from responses in severe disasters. Subsequent peer review of the initial version of the standards provided additional perspective from a broader base of stakeholders. Later piloting of the document in verification of type 2 EMTs revealed team perception of and response to the standards as well as issues encountered in their achievement and demonstration. Feedback was integrated into the final, published version of the document, strengthening its validity and facilitating operational implementation.

Another consideration addressed in the development of the standards was the significant variability in national rehabilitation capacity amongst countries. The WHO EMT Initiative is a global initiative, meaning the standards apply for all low-, middle- and high-income countries seeking verification. National rehabilitation capacity around the world is hugely variable, with some countries having limited to no professional rehabilitation workforce or services 21^, ^22^, ^23. Rehabilitation in acute care settings in these low-resource countries is particularly deficient. The working group attempted to develop standards which would ensure an acceptable level of care and would be feasibly achieved, with or without external support (such as from an expatriate nongovernmental organization), for countries with low rehabilitation capacity.

The relatively low rehabilitation capacity in many countries challenges the acceptance and implementation of the standards in these countries. Some teams have been reluctant to bear the necessary financial and logistical burden of integrating the standards into their service capacity 21^, ^22^, ^23. In these cases, strong advocacy and guidance from EMT mentors is of particular importance, as they are well placed to educate teams on the necessity of early access to rehabilitation for achieving optimum patient outcomes for patients, and to guide teams in addressing the logistical and cost challenges of implementation. Ongoing dissemination of the EMT initiative communication materials will further sensitize teams to the role and benefits of rehabilitation in emergency response over time.

While this protocol report focuses on the development and implementation of the standards from an EMT perspective, a significant implication to note for individual rehabilitation professionals is their increasing participation on teams, ideally those which adhere to the standards. It is fundamental that rehabilitation professionals who desire to respond in emergencies are aware of the EMT initiative and seek out government or nongovernmental teams to integrate into. As with many health professionals, rehabilitation providers have historically responded as individuals or in ad hoc groups with varying collaboration with the affected government/coordinating body resulting in highly variable, low-quality care. The standards aim to curtail this approach by promoting the integration of rehabilitation within EMTs and specialized care teams. Reference guidance documents such as Responding internationally to disasters: A do’s and don’ts guide for rehabilitation professionals and the World Confederation for Physical Therapy report, The role of physical therapists in disaster response, encourage rehabilitation providers to integrate into EMTs 24^, ^25.

## **Current and next steps**

Additional WHO EMT initiative activities to support EMTs in achieving the standards include:

1. Strengthening coordination of EMTs to include rehabilitation. This enhanced coordination will help maximize the impact of available rehabilitation capacity by directing resources and facilitating smooth transfer of patients. Developing standard operating procedures for the coordination of rehabilitation in EMTs will support this activity, ensuring that the lessons learnt and best practices from rehabilitation coordination in past emergencies (such as Nepal) are translated into an actionable format for future emergencies.
2. Building a global network of rehabilitation advisers. This network will support the mentorship of EMTs, especially in countries with low rehabilitation capacity, and members will act as advisers in the EMT coordination cell in the affected country during the disaster response.
3. Integrating the standards and recommendations for rehabilitation in other ‘specialized care teams’, such as those for burns and spinal cord injury, which have rehabilitation components to their care. These standards will specify the rehabilitation expertise and materials required for team deployment and would be used for verification.

Moreover, building rehabilitation into national emergency preparedness plans is necessary to ensure contingency plans are in place and to optimize service delivery. Continued health system strengthening is also essential for ensuring continuity of quality rehabilitation for those with long-term impairments sustained in an emergency.

## Conclusion

The WHO EMT Initiative aims to improve the professionalization and quality of emergency medical response through improved standardization, coordination and accountability. The rehabilitation standards support these aims, by addressing by integrating delivery of rehabilitation into the acute emergency response capacity of EMTs. Required for EMT verification, the standards fill a significant in the scope and quality of care EMTs have historically provided. The standards also emphasize the importance of continuity of care and long-term functional outcomes by beneficiaries. A need exists for further awareness raising of standards, the role and benefits of rehabilitation in the context of acute emergency medical response, and the necessity of strengthening health system to improve national rehabilitation capacity.

## Data Availability

All relevant data are within the paper.

## Competing Interests

The authors have declared that no competing interests exist.

*Jody-Anne Mills and Ian Norton are staff members of the World Health Organization in Geneva. They alone are responsible for the views expressed in this publication and they do not necessarily represent the decisions or policies of any third party.*

## Corresponding Author

Jody-Anne Mills (millsj@who.int)

## References

[1] Norton I, Von Schreeb J, Aitken P, Herard P, Lajolo C. Classification and minimum standards for foreign medical teams in sudden onset disasters. Geneva: WHO Press; 2013.

[2] World Health Organization. Rehabilitation in health systems. Geneva, Switzerland: WHo Press; 2017.

[3] Choi JH, Jakob M, Stapf C, Marshall RS, Hartmann A, Mast H. Multimodal early rehabilitation and predictors of outcome in survivors of severe traumatic brain injury. J Trauma. 2008 Nov;65(5):1028-35. PubMed PMID:19001970. 10.1097/TA.0b013e31815eba9b 19001970

[4] Petruseviciene D, Krisciūnas A. Evaluation of activity and effectiveness of occupational therapy in stroke patients at the early stage of rehabilitation. Medicina (Kaunas). 2008;44(3):216-24. PubMed PMID:18413989. 18413989

[5] Scivoletto G, Morganti B, Molinari M. Early versus delayed inpatient spinal cord injury rehabilitation: an Italian study. Arch Phys Med Rehabil. 2005 Mar;86(3):512-6. PubMed PMID:15759237. 10.1016/j.apmr.2004.05.021 15759237

[6] Nielsen PR, Andreasen J, Asmussen M, Tønnesen H. Costs and quality of life for prehabilitation and early rehabilitation after surgery of the lumbar spine. BMC Health Serv Res. 2008 Oct 9;8:209. PubMed PMID:18842157. 10.1186/1472-6963-8-209 18842157 PMC2586633

[7] Li Y, Reinhardt JD, Gosney JE, Zhang X, Hu X, Chen S, Ding M, Li J. Evaluation of functional outcomes of physical rehabilitation and medical complications in spinal cord injury victims of the Sichuan earthquake. J Rehabil Med. 2012 Jun;44(7):534-40. PubMed PMID:22674233. 10.2340/16501977-1005 22674233

[8] World Health Organization, The World Bank. World report on disability. Geneva, Switzerland: WHO Press; 2011.

[9] Landry MD, O'Connell C, Tardif G, Burns A. Post-earthquake Haiti: the critical role for rehabilitation services following a humanitarian crisis. Disabil Rehabil. 2010;32(19):1616-8. PubMed PMID:20594036. 10.3109/09638288.2010.500345 20594036

[10] Iezzoni LI, Ronan LJ. Disability legacy of the Haitian earthquake. Ann Intern Med. 2010 Jun 15;152(12):812-4. PubMed PMID:20231547. 10.7326/0003-4819-152-12-201006150-00234 20231547

[11] Rathore FA, Farooq F, Muzammil S, New PW, Ahmad N, Haig AJ. Spinal cord injury management and rehabilitation: highlights and shortcomings from the 2005 earthquake in Pakistan. Arch Phys Med Rehabil. 2008 Mar;89(3):579-85. PubMed PMID:18295642. 10.1016/j.apmr.2007.09.027 18295642

[12] Rathore FA, Gosney JE, Reinhardt JD, Haig AJ, Li J, DeLisa JA. Medical rehabilitation after natural disasters: why, when, and how? Arch Phys Med Rehabil. 2012 Oct;93(10):1875-81. PubMed PMID:22676904. 10.1016/j.apmr.2012.05.018 22676904

[13] Reinhardt JD, Li J, Gosney J, Rathore FA, Haig AJ, Marx M, DeLisa JA. Disability and health-related rehabilitation in international disaster relief. Glob Health Action. 2011;4:7191. PubMed PMID:21866223. 10.3402/gha.v4i0.7191 21866223 PMC3160807

[14] Chackungal S, Nickerson JW, Knowlton LM, Black L, Burkle FM, Casey K, Crandell D, Demey D, Di Giacomo L, Dohlman L, Goldstein J, Gosney JE Jr, Ikeda K, Linden A, Mullaly CM, O'Connell C, Redmond AD, Richards A, Rufsvold R, Santos AL, Skelton T, McQueen K. Best practice guidelines on surgical response in disasters and humanitarian emergencies: report of the 2011 Humanitarian Action Summit Working Group on Surgical Issues within the Humanitarian Space. Prehosp Disaster Med. 2011 Dec;26(6):429-37. PubMed PMID:22475370. 10.1017/S1049023X12000064 22475370

[15] Knowlton LM, Gosney JE, Chackungal S, Altschuler E, Black L, Burkle FM Jr, Casey K, Crandell D, Demey D, Di Giacomo L, Dohlman L, Goldstein J, Gosselin R, Ikeda K, Le Roy A, Linden A, Mullaly CM, Nickerson J, O'Connell C, Redmond AD, Richards A, Rufsvold R, Santos AL, Skelton T, McQueen K. Consensus statements regarding the multidisciplinary care of limb amputation patients in disasters or humanitarian emergencies: report of the 2011 Humanitarian Action Summit Surgical Working Group on amputations following disasters or conflict. Prehosp Disaster Med. 2011 Dec;26(6):438-48. PubMed PMID:22559308. 10.1017/S1049023X12000076 22559308

[16] Burns AS, O'Connell C, Rathore F. Meeting the challenges of spinal cord injury care following sudden onset disaster: lessons learned. J Rehabil Med. 2012 May;44(5):414-20. PubMed PMID:22549649. 10.2340/16501977-0974 22549649

[17] Zhang X, Hu XR, Reinhardt JD, Zhu HJ, Gosney JE, Liu SG, Li J. Functional outcomes and health-related quality of life in fracture victims 27 months after the Sichuan earthquake. J Rehabil Med. 2012 Mar;44(3):206-9. PubMed PMID:22367105. 10.2340/16501977-0945 22367105

[18] World Health Organization. Emergency medical teams: Minimum technical standards and recommendations for rehabilitation Geneva: WHO Press; 2016. 10.1371/currents.dis.76fd9ebfd8689469452cc8c0c0d7cdce PMC6050053 30050723

[19] Bausch DG. Sequelae after Ebola virus disease: even when it's over it's not over. Lancet Infect Dis. 2015 Aug;15(8):865-6. PubMed PMID:25910638. 10.1016/S1473-3099(15)70165-9 25910638

[20] World Health Organization. WHO global disability action plan 2014-2021. Better health for all people with disability. Geneva, Switzerland: WHO Press; 2015.

[21] Agho AO, John EB. Occupational therapy and physiotherapy education and workforce in Anglophone sub-Saharan Africa countries. Hum Resour Health. 2017 Jun 12;15(1):37. PubMed PMID:28606103. 10.1186/s12960-017-0212-5 28606103 PMC5469184

[22] Gupta N, Castillo-Laborde C, Landry MD. Health-related rehabilitation services: assessing the global supply of and need for human resources. BMC Health Serv Res. 2011 Oct 17;11:276. PubMed PMID:22004560. 10.1186/1472-6963-11-276 22004560 PMC3207892

[23] Jesus TS, Landry MD, Dussault G, Fronteira I. Human resources for health (and rehabilitation): Six Rehab-Workforce Challenges for the century. Hum Resour Health. 2017 Jan 23;15(1):8. PubMed PMID:28114960. 10.1186/s12960-017-0182-7 28114960 PMC5259954

[24] Skelton P, Foo W. Responding internationally to disasters: A do's and don'ts guide for rehabilitation professionals. London, United Kingdom: Handicap International; 2016.

[25] World Confederation for Physical Therapy. WCPT report: The role of physical therapists in disaster management. London: World Confederation for Physical Therapy, 2016.

